# Characteristics of patients with atrial fibrillation prescribed edoxaban in Belgium and the Netherlands: insights from the ETNA-AF-Europe study

**DOI:** 10.1007/s12471-020-01518-7

**Published:** 2021-01-07

**Authors:** T. A. C. de Vries, M. E. W. Hemels, F. Cools, H. J. G. M. Crijns, L. Yperzeele, P. Vanacker, I. Blankoff, P. Lancellotti, G. H. Mairesse, A. de Veer, R. Casado Arroyo, E. Catez, M. de Pauw, T. Vanassche, C. de Asmundis, P. Kirchhof, R. De Caterina, J. R. de Groot, the ETNA-AF-Europe principal investigators from Belgium and the Netherlands*

**Affiliations:** 1 Department of Cardiology, Rijnstate Hospital, Arnhem, The Netherlands; 2 Department of Cardiology, Amsterdam Medical Centres/University of Amsterdam, Amsterdam, The Netherlands; 3 Department of Cardiology, Radboud University Medical Centre, Nijmegen, The Netherlands; 4 Department of Cardiology, General Hospital Klinieken Noord-Antwerpen, Brasschaat, Belgium; 5 Department of Cardiology, Maastricht University Medical Centre+, Maastricht, The Netherlands; 6 Department of Neurosurgery, University Hospital Antwerp, Antwerp, Belgium; 7 Department of Neurology, General Hospital Groeninge, Kortrijk, Belgium; 8 Department of Cardiology, Civil Hospital Marie Curie, Charleroi, Belgium; 9 Department of Cardiology, University Hospital of Liège, Liège, Belgium; 10 Department of Cardiology, Cliniques du Sud-Luxembourg, Arlon, Belgium; 11 Department of Cardiology, St. Antonius Hospital, Nieuwegein, The Netherlands; 12 Department of Cardiology, Hospital Erasme, Anderlecht, Belgium; 13 Department of Cardiology, Brugmann University Hospital, Brussels, Belgium; 14 Department of Cardiology, Ghent University Hospital, Ghent, Belgium; 15 Department of Cardiology, Leuven University Hospital, Leuven, Belgium; 16 Department of Cardiology, University Hospital Brussels, Brussels, Belgium; 17 Institute of Cardiovascular Sciences, University of Birmingham, Birmingham, UK; 18 The Atrial Fibrillation NETwork (AFNET), Münster, Germany; 19 Department of Cardiology, University of Pisa, Pisa, Italy

**Keywords:** Anticoagulants, Haemorrhage, Off-label use, Clinical trial, phase IV, Stroke, Thromboembolism

## Abstract

**Background:**

Studies on the use of non-vitamin K antagonist oral anticoagulants in unselected patients with atrial fibrillation (AF) show that clinical characteristics and dosing practices differ per region, but lack data on edoxaban.

**Methods:**

With data from Edoxaban Treatment in routiNe clinical prActice for patients with AF in Europe (ETNA-AF-Europe), a large prospective observational study, we compared clinical characteristics (including the dose reduction criteria for edoxaban: creatinine clearance 15–50 ml/min, weight ≤60 kg, and/or use of strong p‑glycoprotein inhibitors) of patients from Belgium and the Netherlands (BeNe) with those from other European countries (OEC).

**Results:**

Of all 13,639 patients in ETNA-AF-Europe, 2579 were from BeNe. BeNe patients were younger than OEC patients (mean age: 72.3 vs 73.9 years), and had lower CHA_2_DS_2_-VASc (mean: 2.8 vs 3.2) and HAS-BLED scores (mean: 2.4 vs 2.6). Patients from BeNe less often had hypertension (61.6% vs 80.4%), and/or diabetes mellitus (17.3% vs 23.1%) than patients from OEC. Moreover, relatively fewer patients in BeNe were prescribed the reduced dose of 30 mg edoxaban (14.8%) than in OEC (25.4%). Overall, edoxaban was dosed according to label in 83.1% of patients. Yet, 30 mg edoxaban was prescribed in the absence of any dose reduction criteria in 36.9% of 30 mg users (5.5% of all patients) in BeNe compared with 35.5% (9.0% of all patients) in OEC.

**Conclusion:**

There were several notable differences between BeNe and OEC regarding clinical characteristics and dosing practices in patients prescribed edoxaban, which are relevant for the local implementation of dose evaluation and optimisation.

**Trial registration:**

NCT02944019; Date of registration 24 October 2016

**Electronic supplementary material:**

The online version of this article (10.1007/s12471-020-01518-7) contains supplementary material, which is available to authorized users.

## Introduction

Several large real-world evidence studies have been performed to study the safety of the non-vitamin K antagonist oral anticoagulants (NOACs) for stroke prevention in unselected patients with atrial fibrillation (AF). These studies confirm that use of NOACs in routine clinical practice is safe and efficacious, but also show that their reduced doses are far more often prescribed than in the randomised controlled trials (RCTs) [1–14]. These and other phase IV studies on the use of NOACs in patients with AF show important differences among geographical regions regarding patient characteristics and prescription patterns [1, 5–10, 12–14].

Such information is crucial to allow healthcare personnel (e.g. physicians, pharmacologists, or policymakers) to more accurately address potential local issues, as well as to translate findings of continental or global studies to our local practices. However, as all these studies included data before or shortly after the approval of edoxaban, such data on this NOAC are scarce [1, 5–10, 12–15].

Edoxaban is a direct factor Xa inhibitor, approved in 2015 for stroke prevention in adult non-valvular AF patients [15]. According to its summary of product characteristics (SmPC), the approved dose is 60 mg once daily (OD), with a dose reduction to 30 mg OD in patients with a creatinine clearance (CrCl) between 15 and 50 ml/min, a body weight ≤60 kg, and/or concomitant use of strong p‑glycoprotein (p-gp) inhibitors, i.e. cyclosporine, dronedarone, erythromycin, and ketoconazole [15].

Recently, the Edoxaban Treatment in routiNe clinical prActice for patients with non-valvular AF in Europe (ETNA-AF-Europe) study (Clinicaltrials.gov: NCT02944019) completed patient enrolment. This registry allows us to determine whether there are also important regional differences in clinical practice for edoxaban [16]. Here, we describe the characteristics of edoxaban users with AF from Belgium and the Netherlands (BeNe) compared to those from other European countries (OEC).

## Methods

The ETNA-AF-Europe registry study is an observational, post-authorisation study in which patients from ten European countries (Austria, Belgium, Germany, Ireland, Italy, the Netherlands, Portugal, Spain, Switzerland, and the United Kingdom) are followed for up to 48 months. All patients with AF, diagnosed by electrical tracing (i.e. electrocardiogram, Holter monitoring, pacemaker or a different implantable device) within the last 12 months, and treated with edoxaban were eligible for inclusion. No explicit exclusion criteria were applied [17].

In addition to standard demographics, data on the history of cardiovascular diseases (e.g. hypertension, prior ischaemic stroke or major bleeding), weight, renal function, and on AF-related therapies (e.g. prior use of anticoagulants, and current use of antiplatelet drugs) were collected. Other details on the methods and design of ETNA-AF-Europe have been reported previously [17].

Although ETNA-AF-Europe is one of the largest phase IV registries on patients with AF to date, we decided that due to modest patient numbers per country, pooling data from two neighbouring countries would be more desirable than to further divide regions with the result of precluding meaningful comparisons.

Based on the above considerations, we extracted data on the baseline characteristics of patients from BeNe that were enrolled in ETNA-AF-Europe; determined the proportional use of 30 mg OD and 60 mg OD edoxaban, and whether dose selection was in accordance with the SmPC (except for concomitant use of p‑gp inhibitors). We then compared these findings with those from OEC to assess for clinically important regional differences.

In line with our rationale to interpret findings from continental or global studies in a regional context, we also compared characteristics of patients observed in ETNA-AF-Europe that were from BeNe with those from the corresponding countries once enrolled in the Effective Anticoagulation With Factor Xa Next Generation in Atrial Fibrillation–Thrombolysis in Myocardial Infarction 48 (ENGAGE-AF-TIMI 48) trial [3].

## Results

### Belgium and the Netherlands compared with other European countries

**Fig. 1:**
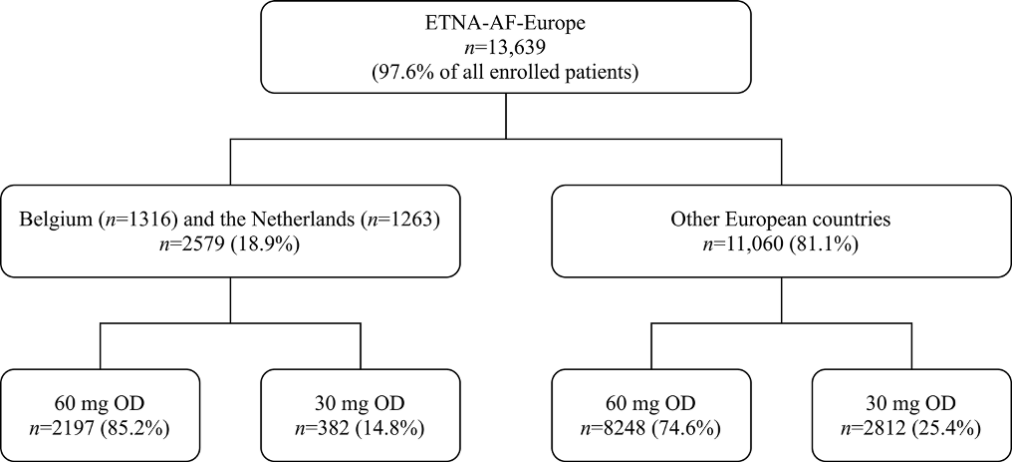
Patient selection. Overview of patient enrolment in the ETNA-AF-Europe registry. *ETNA-AF Europe* Edoxaban Treatment in routiNe clinical prActice for patients with non-valvular Atrial Fibrillation in Europe, *OD* once daily. ^a^Austria, Germany, Ireland, Italy, Portugal, Spain, Switzerland, and the United Kingdom

A total of 13,639 (97.6% of the 13,980 enrolled patients in ETNA-AF-Europe) were included in our analyses. Of these, 2579 (18.9%) were from BeNe: 1316 (9.6%) from Belgium and 1263 (9.3%) from the Netherlands (Fig. 1). The baseline demographics and clinical characteristics for patients enrolled throughout all OEC, and those in BeNe, stratified by dose of edoxaban, are summarised in Tab. 1.

**Table 1:** Patient characteristics

	Belgium and the Netherlands			Other European countries		
Characteristics	Overall (*n* = 2579)	60 mg OD (*n* = 2197)	30 mg OD (*n* = 382)	Overall (*n* = 11,060)	60 mg OD (*n* = 8248)	30 mg OD (*n* = 2812)
30 mg users	382 (14.8)	0 (0.0)	382 (100.0)	2812 (25.4)	0 (0.0)	2812 (100.0)
Male	1514 (58.8)	1356 (61.8)	158 (41.4)	6193 (56.0)	4961 (60.1)	1232 (43.8)
Age (years)	72.3 ± 9.1	71.2 ± 8.7	78.9 ± 8.4	73.9 ± 9.6	71.9 ± 9.3	79.7 ± 7.8
*Age subgroups (years)*						
– <65	439 (17.0)	422 (19.2)	17 (4.5)	1658 (15.0)	1541 (18.7)	117 (4.2)
– 65–74	1056 (41.0)	969 (44.1)	87 (22.8)	3550 (32.1)	3062 (37.1)	488 (17.4)
– 75–84	882 (34.2)	704 (32.1)	178 (46.6)	4618 (41.8)	3191 (38.7)	1427 (50.8)
– ≥85	200 (7.8)	100 (4.6)	100 (26.2)	1232 (11.1)	453 (5.5)	779 (27.7)
Weight (kg)	82.3 ± 17.4	84.1 ± 16.9	72.3 ± 17.2	80.7 ± 17.3	83.4 ± 16.7	72.9 ± 16.6
BMI (kg/m^2^)	28.0 ± 5.1	28.3 ± 5.0	26.1 ± 4.9	28.1 ± 5.2	28.7 ± 5.1	26.6 ± 5.1
*Blood pressure (mm Hg)*						
– Systolic	136.3 ± 20.3	135.9 ± 20.2	138.1 ± 21.0	132.8 ± 17.4	133.1 ± 17.1	131.9 ± 18.0
– Diastolic	78.8 ± 12.4	79.3 ± 12.3	76.0 ± 12.1	78.2 ± 10.5	78.9 ± 10.5	76.2 ± 10.4
Active smokers	208 (8.1)	184 (8.4)	24 (6.3)	651 (5.9)	535 (6.5)	116 (4.1)
No alcohol use	629 (24.4)	501 (22.8)	128 (33.5)	5483 (49.7)	3880 (47.1)	1603 (57.0)
CrCl^a^ (ml/min)	78.4 ± 29.7	83.7 ± 28.2	49.6 ± 19.3	73.5 ± 30.6	81.9 ± 29.5	50.3 ± 19.8
*CrCl*^a^* subgroups (ml/min)*						
– <15	0 (0.0)	0 (0.0)	0 (0.0)	2 (0.0)	0 (0.0)	2 (0.1)
– 15–30	45 (2.0)	9 (0.5)	36 (10.5)	250 (2.6)	30 (0.4)	220 (8.7)
– 30–50	276 (12.5)	113 (6.0)	163 (47.5)	1899 (19.8)	585 (8.3)	1314 (51.7)
– 50–80	953 (43.0)	830 (44.4)	123 (35.9)	4095 (42.7)	3286 (46.6)	809 (31.8)
– ≥80	940 (42.5)	919 (49.1)	21 (6.1)	3349 (34.9)	3152 (44.7)	197 (7.7)
Renal disease	360 (14.0)	206 (9.4)	154 (40.3)	3329 (30.1)	1836 (22.3)	1493 (53.1)
CHADS_2_^a^	1.5 ± 1.1	1.4 ± 1.1	2.0 ± 1.1	1.8 ± 1.1	1.7 ± 1.0	2.2 ± 1.0
CHA_2_DS_2_-VASc^a^	2.8 ± 1.4	2.6 ± 1.4	3.6 ± 1.3	3.2 ± 1.4	3.0 ± 1.4	3.8 ± 1.3
– Score of 0	99 (3.8)	97 (4.4)	2 (0.5)	211 (1.9)	203 (2.5)	8 (0.3)
– Score of ≥1	2480 (96.2)	2100 (95.6)	380 (99.5)	10,849 (98.1)	8045 (97.5)	2804 (99.7)
HAS-BLED^a^	2.4 ± 1.2	2.3 ± 1.1	2.9 ± 1.2	2.6 ± 1.1	2.5 ± 1.1	3.0 ± 1.1
*Frailty*^b^						
– Yes	153 (5.9)	80 (3.6)	73 (19.1)	1289 (11.7)	556 (6.8)	733 (26.1)
– No	2184 (84.7)	1915 (87.2)	269 (70.4)	9076 (82.2)	7192 (87.4)	1884 (67.0)
– Unknown	240 (9.3)	200 (9.1)	40 (10.5)	677 (6.1)	482 (5.9)	195 (6.9)
*History of cardiovascular diseases*						
– Hypertension	1589 (61.6)	1335 (60.8)	254 (66.5)	8891 (80.4)	6588 (79.9)	2303 (81.9)
– CHF	109 (4.2)	73 (3.3)	36 (9.4)	690 (6.2)	422 (5.1)	268 (9.5)
– MI	130 (5.0)	94 (4.3)	36 (9.4)	454 (4.1)	293 (3.6)	161 (5.7)
– Angina pectoris	47 (1.8)	37 (1.7)	10 (2.6)	154 (1.4)	97 (1.2)	57 (2.0)
– Valvular disease	345 (13.4)	252 (11.5)	93 (24.3)	2081 (18.8)	1445 (17.5)	636 (22.6)
– PAD	93 (3.6)	79 (3.6)	14 (3.7)	365 (3.3)	214 (2.6)	151 (5.4)
– DM	445 (17.3)	360 (16.4)	85 (22.3)	2551 (23.1)	1782 (21.6)	769 (27.3)
*History of stroke*						
– Ischaemic	154 (6.0)	121 (5.5)	33 (8.6)	662 (6.0)	472 (5.7)	190 (6.8)
– Cryptogenic	10 (0.4)	9 (0.4)	1 (0.3)	71 (0.6)	44 (0.5)	27 (1.0)
– TIA	129 (5.0)	105 (4.8)	24 (6.3)	334 (3.0)	238 (2.9)	96 (3.4)
*History of bleeding*						
– Gastrointestinal^c^	19 (0.7)	14 (0.6)	5 (1.3)	89 (0.8)	39 (0.5)	50 (1.8)
– Major	31 (1.2)	24 (1.1)	7 (1.8)	102 (0.9)	58 (0.7)	44 (1.6)
– Intracranial	19 (0.7)	16 (0.7)	3 (0.8)	48 (0.4)	30 (0.4)	18 (0.6)
*Atrial fibrillation type*						
– Paroxysmal	1668 (64.9)	1417 (64.7)	251 (65.7)	5612 (50.9)	4261 (51.9)	1351 (48.1)
– Persistent	585 (22.8)	500 (22.8)	85 (22.3)	2724 (24.7)	2131 (25.9)	593 (21.1)
– Long-standing persistent	30 (1.2)	27 (1.2)	3 (0.8)	303 (2.7)	217 (2.6)	86 (3.1)
– Permanent	228 (11.2)	245 (11.2)	43 (11.3)	2382 (21.6)	1606 (19.5)	776 (27.7)
*Burden of atrial fibrillation*						
– Symptomatic	1479 (57.4)	1285 (58.6)	194 (50.8)	5776 (52.3)	4404 (53.5)	1372 (48.8)
– Asymptomatic	856 (33.2)	714 (32.5)	142 (37.2)	3762 (34.1)	2758 (33.5)	1004 (35.7)
– Unknown	241 (9.4)	195 (8.9)	46 (12.0)	1500 (13.6)	1066 (13.0)	434 (15.4)
*Time since the diagnosis of atrial fibrillation (months)*						
– Median (IQR)	1.9 (0.3; 27.5)	1.9 (0.3; 28.1)	2.0 (0.2; 20.1)	5.5 (0.5; 29.7)	4.8 (0.4; 26.3)	7.5 (0.8; 39.1)
*Previous (not current) use of atrial fibrillation relevant medication*						
– VKA	540 (20.9)	469 (21.3)	71 (18.6)	1781 (16.1)	1238 (15.0)	543 (19.3)
– NOAC (other)	174 (6.7)	136 (6.2)	38 (9.9)	924 (8.4)	588 (7.1)	336 (11.9)
– Rate- or rhythm	238 (9.2)	201 (9.1)	37 (9.7)	445 (4.0)	327 (4.0)	118 (4.2)
– Antiplatelets	498 (19.3)	414 (18.8)	84 (22.0)	1572 (14.2)	1137 (13.8)	435 (15.5)
*Number of dose adjustment criteria*^d^						
– 0	2127 (82.5)	1986 (90.4)	141 (36.9)	8293 (75.0)	7295 (88.4)	998 (35.5)
– ≥1	452 (17.5)	211 (9.6)	241 (63.1)	2767 (25.0)	953 (11.6)	1814 (64.5)
*Dose adjustment criteria*^d^						
– CrCl^a^ ≤50 ml/min	321 (14.5)	122 (6.5)	199 (58.0)	2151 (22.4)	615 (8.7)	1536 (60.4)
– Weight ≤60 kg	211 (8.9)	98 (4.8)	113 (31.7)	1158 (10.7)	409 (5.1)	749 (27.2)

This table summarises the clinical characteristics of patients from Belgium or the Netherlands that were enrolled in ETNA-AF-Europe, and of those from the other European countries participating in the registry. Values are number (%) or mean ± SD unless stated otherwise

OD once daily; SD standard deviation; BMI body mass index; mm Hg millimetre of mercury; CrCl creatinine clearance; CHADS_2_ congestive heart failure, hypertension, age ≥75 years, diabetes mellitus, stroke (double weight); CHA_2_DS_2_-VASc congestive heart failure, hypertension, age ≥75 years (double weight), diabetes mellitus, stroke (double weight), vascular disease, age 65–74 years, sex category; HAS-BLED hypertension, abnormal renal and liver function, stroke, bleeding, labile International Normalised Ratio, elderly, drugs or alcohol; NR not reported; CHF chronic heart failure; MI myocardial infarction; PAD peripheral artery disease; DM diabetes mellitus; TIA transient ischaemic attack; IQR interquartile range; VKA vitamin K antagonist; NOAC non-vitamin K antagonist oral anticoagulant; ETNA-AF-Europe Edoxaban Treatment in routiNe clinical prActice for patients with non-valvular Atrial Fibrillation in Europe

^a^Some parameters were reported by the investigators as well as recalculated based on data reported by the investigators. Presented values are those that were recalculated

^b^There was no specific definition for frailty; it was left to the discretion of the physician to categorise a patient as frail

^c^Composite of major bleeding and clinically relevant non-major bleeding

^d^Not including concomitant use of p‑glycoprotein inhibitors for which dose reduction is required according to the summary of product characteristics of edoxaban

**Fig. 2:**
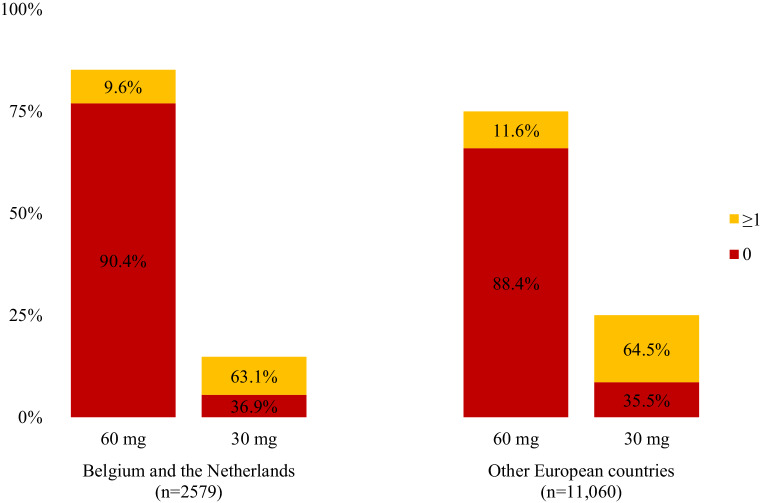
Number of dose reduction criteria. The distribution of the number of dose reduction criteria by dose in Belgium and the Netherlands compared with in other European countries. The y‑axis and the columns illustrate the use of 60 mg edoxaban relative to that of 30 mg for each region. The percentages inside the columns show the distribution of the number of dose reduction criteria per dose of edoxaban

Overall, 86.4% and 82.4% of patients in BeNe and OEC, respectively, were treated with edoxaban at doses conforming to the SmPC. Relatively fewer patients in BeNe compared to OEC were treated with the reduced dose of edoxaban (14.8% vs 25.4%), but the distribution of 30 mg prescriptions in the absence of any dose reduction criteria was similar in both regions: 36.9% of 30 mg users (5.5% of all patients) in BeNe, compared with 35.5% (9.0% of all patients) in OEC (Fig. 2).

**Fig. 3:**
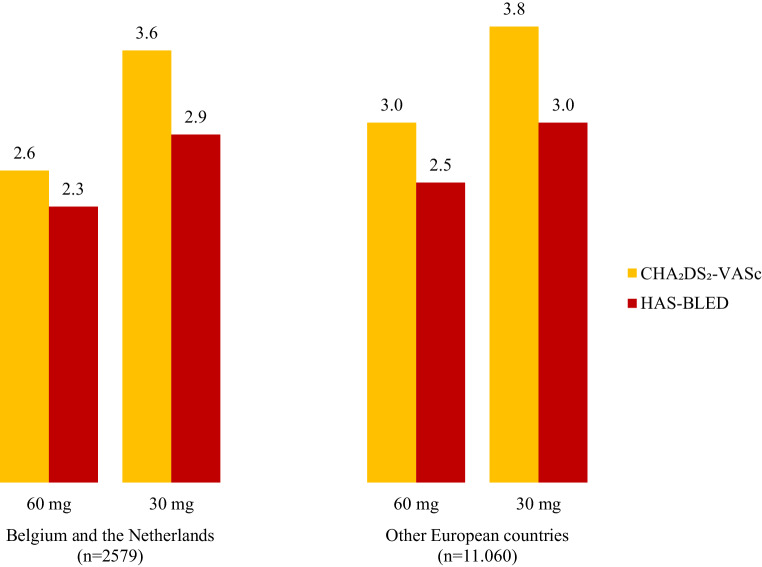
Annual risk scores. The mean CHA_2_DS_2_-VASc and HAS-BLED scores by dose in Belgium and the Netherlands compared with in other European countries. *CHA*_*2*_*DS*_*2*_*-VASc* congestive heart failure, hypertension, age ≥75 years (double weight), diabetes mellitus, stroke (double weight), vascular disease, age 65–74 years, sex category, *HAS-BLED* hypertension, abnormal renal and liver function, stroke, bleeding, labile International Normalised Ratio, elderly, drugs or alcohol

Mean CHA_2_DS_2_-VASc scores were 2.6 for 60 mg users and 3.6 for 30 mg users in BeNe, compared with 3.0 and 3.8, respectively, in OEC. Mean HAS-BLED scores were 2.3 for patients on standard dose edoxaban and 2.9 for those on the reduced dose in BeNe, compared with 2.5 and 3.0, respectively, in OEC (Fig. 3). Overall, in patients from BeNe, a history of cardiovascular disease was less prevalent than in OEC, in particular hypertension (61.6% vs 80.4%) and diabetes mellitus (17.3% vs 23.1%). In contrast, prior ischaemic events were more often reported in BeNe compared with in OEC.

Physicians from BeNe less often described their patients as frail compared with those from OEC (5.9% versus 11.7%). This trend was prevalent in both dosing groups: in BeNe 19.1% of patients on 30 mg were considered frail, and 3.6% of those on 60 mg, compared with 26.1% and 6.8%, respectively, in OEC.

The current type of AF also differed between the two regions, which was most marked for the 30 mg group. Thus, for BeNe patients prescribed 30 mg of edoxaban, 65.7% had paroxysmal AF and 11.3% permanent AF, compared with 48.1% and 27.7%, respectively, of patients prescribed 30 mg in OEC. For the patients on 60 mg from BeNe, paroxysmal AF was reported in 64.7% of cases and permanent AF in 11.2%, relative to 51.8% and 19.5%, respectively, in the other regions of Europe.

### Belgium and the Netherlands: clinical practice versus the phase III trial

The baseline characteristics of patients from BeNe enrolled in the ETNA-AF-Europe registry and those from the corresponding countries included in the ENGAGE-AF-TIMI 48 trial are summarised in Tab. 2.

**Table 2:** Belgium and the Netherlands: unselected versus selected patients

Characteristics	ETNA-AF-Europe (*n* = 2579)	ENGAGE-AF-TIMI 48 (*n* = 302)
Male	1514 (58.8)	196 (64.9)
Age (years)	72.3 ± 9.1	71.5 ± 7.6
*Age subgroups (years)*		
– <65	439 (17.0)	53 (17.5)
– 65–74	1056 (41.0)	102 (33.8)
– ≥75	1082 (42.0)	147 (48.7)
Body weight (kg)	82.3 ± 17.4	84.6 ± 16.5
– ≤60 kg	211 (8.9)	11 (3.6)
BMI (kg/m^2^)	28.0 ± 5.1	28.8 ± 5.0
CrCl^a^ (ml/min)	78.4 ± 29.7	73.4 ± 25.7
*CrCl subgroups*^a^* (ml/min)*		
– <15	0 (0.0)	1 (0.3)
– 15–30	45 (2.0)	2 (0.7)
– 30–50	276 (12.5)	51 (17.1)
– 50–80	953 (43.0)	147 (49.3)
– ≥80	940 (42.5)	97 (32.6)
CHADS_2_^a^	1.5 ± 1.1	2.7 ± 0.9
CHA_2_DS_2_-VASc^a^	2.8 ± 1.4	4.2 ± 1.3
HAS-BLED^a^	2.4 ± 1.2	1.6 ± 1.0
*History of cardiovascular disease*		
– Hypertension	1589 (61.6)	252 (83.4)
– Congestive heart failure	109 (4.2)	94 (31.1)
– Myocardial infarction	130 (5.0)	40 (13.2)
– Diabetes mellitus	445 (17.3)	127 (42.1)
– Ischaemic stroke	154 (6.0)	49 (16.2)
– Transient ischaemic attack	129 (5.0)	52 (17.2)
*Atrial fibrillation type*		
– Paroxysmal	1668 (64.9)	117 (38.7)
– Persistent	585 (22.8)	77 (25.5)
– Long-standing persistent or permanent	318 (12.4)	108 (35.8)

This table summarises the clinical characteristics of patients from Belgium or the Netherlands that were enrolled in ETNA-AF-Europe and of those that were included in the ENGAGE-AF-TIMI 48 randomised trial. All values are number (%) or mean ± standard deviation

ETNA-AF-Europe Edoxaban Treatment in routiNe clinical prActice for patients with non-valvular Atrial Fibrillation in Europe; ENGAGE AF-TIMI 48 Effective Anticoagulation with Factor Xa Next Generation in Atrial Fibrillation—Thrombolysis in Myocardial Infarction 48; SD standard deviation; BMI body mass index; CrCl creatinine clearance; CHADS_2_ congestive heart failure, hypertension, age ≥75 years, diabetes mellitus, stroke (double weight); CHA_2_DS_2_-VASc congestive heart failure, hypertension, age ≥75 years (double weight), diabetes mellitus, stroke (double weight), vascular disease, age 65–74 years, sex category; HAS-BLED hypertension, abnormal renal and liver function, stroke, bleeding, labile International Normalised Ratio, elderly, drugs or alcohol

^a^Some parameters were reported by the investigators as well as recalculated based on data reported by the investigators. Presented values are those that were recalculated

Overall, 302 patients from BeNe were enrolled in the RCT (vs 2579 in the registry). CHA_2_DS_2_-VASc scores were much lower in the registry than in the RCT (mean: 2.8 vs 4.2), as were the rates of prior cardiovascular diseases. Conversely, HAS-BLED scores were higher in ETNA-AF-Europe than in ENGAGE-AF-TIMI 48 (mean: 2.4 vs 1.6).

## Discussion

Our analyses on differences among geographical regions regarding patient characteristics and prescription patterns using baseline data from ETNA-AF-Europe show three important observations. First, the 30 mg dose of edoxaban was used much more frequently in OEC than in BeNe. Second, compared with those from OEC, patients from BeNe had slightly better overall prognostic characteristics. Lastly, patients from BeNe once enrolled in ENGAGE-AF-TIMI 48 generally had characteristics that put them at much higher baseline risks of stroke than those from our cohort.

### Less use of 30 mg edoxaban and better prognostic characteristics in Belgium and the Netherlands compared with in other European countries

Our results show that the 30 mg dose was used far less frequently in BeNe than in the OEC. However, rates of use of the 30 mg dose in the absence of any dose reduction criteria were similar in both regions, which can only be explained by relatively fewer patients in BeNe than in OEC with criteria for dose reduction: 14.5% in BeNe had a CrCl ≤50 ml/min compared with 22.4% in OEC; and 8.9% and 10.7% had a body weight ≤60 kg in BeNe and OEC, respectively. Often, such characteristics are related to other comorbidities, and consequently also to worse prognostic characteristics. That patients in BeNe, in fact, had better prognostic characteristics than those in OEC is evidenced by three observations.

First, a history of hypertension and diabetes mellitus was less common in BeNe, resulting in slightly lower mean CHA_2_DS_2_-VASc (−0.4) and HAS-BLED (−0.2) scores. Second, patients were less often considered frail in BeNe than in OEC (−5.8 percentage points). Lastly, more patients had paroxysmal AF in BeNe than in OEC (+14.0 percentage points), whereas permanent AF (−10.4 percentage points) was less often reported.

Aside from the role of chance, there are two potential explanations for the differences in clinical characteristics between BeNe and OEC: (a) other prescription preferences; and/or (b) other intrinsic risks.

### Differences in prescription preferences

It might be that physicians in BeNe prefer to prescribe edoxaban to AF patients with better risk profiles, and therefore the other NOACs to patients with worse profiles, and that this trend is less often observed in OEC (or perhaps not at all). For example, more patients in BeNe (3.8%) had a CHA_2_DS_2_-VASc of zero than in OEC (1.9%). This suggests that in our clinical practice edoxaban is more often prescribed for an upcoming or prior electrocardioversion or catheter ablation, and not for chronic stroke prevention, than in the other regions of Europe.

Whether this notion is correct, and whether there are truly other subgroups in which physicians from BeNe relative to those from other regions of Europe prefer to initiate edoxaban over the other NOACs (or vice versa) is still unknown. Some have addressed patterns of NOAC use in BeNe, but were unable to include data for edoxaban [18–20]. Future studies that include all NOACs and address these issues are needed.

### Differences between populations

To our knowledge, no study has compared clinical characteristics of patients in either Belgium or the Netherlands prescribed one of the NOACs with those from OEC. Yet, when comparing the BeNe cohort with those used in other observational studies on NOACs, BeNe patients did not differ much with regard to age and CHA_2_DS_2_-VASc compared to those from Norway (mean age: 70.8–74.5 years; mean CHA_2_DS_2_-VASc: 2.5–2.9) [5], Scotland (mean age: 71.1–74.8 years; mean CHA_2_DS_2_-VASc: 2.5–3.0) [10], and the United Kingdom (mean age: 74.4–76.6 years; CHA_2_DS_2_-VASc: unknown) [14]. However, two studies on German and French patients with AF showed notably higher mean CHA_2_DS_2_-VASc scores compared with our cohort (3.7 vs 3.5–3.9 vs 2.8, respectively) [6, 8]. More studies on regional differences are needed to determine whether BeNe patients treated with oral anticoagulants are healthier than their peers from OEC.

### Higher stroke risks in the randomised trial than in clinical practice

Our comparisons illustrate that relative to Belgian and Dutch patients prescribed edoxaban in ETNA-AF-Europe, those from the corresponding countries in ENGAGE-AF-TIMI 48 had much higher mean CHA_2_DS_2_-VASc scores (+1.4), and much more often a history of any of the reported cardiovascular diseases (1.4- to 7.4-fold). Yet, our analyses also indicate that, compared with in the RCT, edoxaban is utilised in more patients with extreme characteristics in BeNe clinical practice, as demonstrated by more patients with a weight ≤60 kg (+5.3 percentage points), a CrCl ≤30 ml/min (+1.0 percentage point), and by overall higher HAS-BLED scores (mean: +0.8).

These observations are likely attributable to differences in patient selection. For example, one of the inclusion criteria for the RCT was a CHADS_2_ score of ≥2 [3], whereas in ETNA-AF-Europe, patients were eligible for inclusion regardless of their baseline stroke risk [17].

Another important observation is that in ENGAGE-AF-TIMI 48 dose reduction criteria were strictly followed [3], whereas about a third of Belgian and Dutch patients in ETNA-AF-Europe on 30 mg edoxaban did not fulfil the criteria for dose reduction. Similar prescription patterns have been reported for the other NOACs [21], which imply that many of these off-label dose selections are not accidental and, instead, suggest that physicians are knowingly opting for the reduced dose. Although it is still unclear what the true effect is of off-label dose reductions in clinical practice, there are signs from both observational and randomised studies that such prescription reduces overall efficacy [2, 3, 22–24].

Thus, the ENGAGE-AF-TIMI 48 trail compared well-managed warfarin with two strategies of edoxaban: 60 mg, or 30 mg in patients with at least one dose reduction criterion; and 30 mg, with a dose reduction to 15 mg. The latter strategy was not approved for clinical use as this arm was associated with 41% more ischaemic strokes than warfarin [3]. A substudy of this trial, using patients in whom edoxaban drug levels were measured (*n* = 6780), showed that those on 30 mg without criteria for dose reduction experienced 43% more ischaemic strokes than those on warfarin. Conversely, 60 mg and dose reduced 30 mg (in the presence of dose reduction criteria) use were associated with a statistically non-significant reduction in ischaemic stroke of 6 and 4%, respectively [25]. Moreover, several descriptive studies on the use of the other NOACs in clinical practice suggest that off-label dose reductions are associated with more thromboembolic events, without a beneficial reduction in bleeding [22–24]. These observations indicate that an important proportion of patients on edoxaban in our clinical practice in BeNe are insufficiently protected against ischaemic stroke. Still, there might be selected patients in whom off-label use of 30 mg edoxaban could be considered instead of 60 mg.

Thus, first, criteria selected for dose reduction in edoxaban were derived from patients included in pre-phase III studies [26], and might therefore not be generalisable to all patients in clinical practice. Second, in addition to those included in the SmPC, there are several other drugs known to increase the drug exposure of edoxaban, such as verapamil, digoxin, quinidine, and amiodarone [26–28]. Especially in patients with a CrCl and/or a body weight just above 50 ml/min and/or 60 kg, respectively, such drugs might be the tipping point from inappropriate to appropriate off-label use of the 30 mg dose. However, considering the prevalent use of off-label 30 mg edoxaban in clinical practice it is likely that many patients do not fall into this category and are, therefore, probably insufficiently protected against ischaemic stroke.

The primary results of the ETNA-AF-Europe registry, which include ischaemic strokes and major bleeds, will answer whether the efficacy and safety of edoxaban as shown in the ENGAGE-AF-TIMI 48 trial also holds true in unselected AF patients. With this data we will be able to determine whether there are signs that off-label prescriptions of edoxaban are harmful.

## Strengths and limitations

The main strengths of our study are that ETNA-AF-Europe is the largest prospective phase IV study on the use of edoxaban for AF in clinical practice to date, with a total of 13,639 patients from ten countries; and that patient enrolment was well distributed among the participating countries [16].

Although the latter observation strengthens the representativeness of our data, it also comes with our most important limitation since we had to arbitrarily pool data from two neighbouring countries due to modest patient numbers per country. Consequently, the regional differences presented in our study might not be completely generalisable to either Belgian or Dutch clinical practice. Even so, we doubt that this limitation has had an important effect on our results and that any differences between these regions would be clinically relevant. Thus, although mere speculation with regard to edoxaban-related care, BeNe have relatively similar patient populations with regard to overall cardiovascular risk profiles and life expectancy, as well as healthcare systems [29].

## Conclusion

With data from the largest phase IV study on edoxaban users with AF to date, we observed several important differences regarding patient characteristics and dose selections between BeNe and OEC. This information adds to the interpretation of the international literature within BeNe routine clinical practice, and is relevant for the local implementation of dose evaluation and optimisation.

## Caption Electronic Supplementary Material

Principal Investigators of ETNA-AF-Europe from Belgium and the Netherlands

## References

[1] Beyer-Westendorf J, Camm AJ, Fox KAA (2019). International longitudinal registry of patients with atrial fibrillation and treated with rivaroxaban: RIVaroxaban evaluation in real life setting (RIVER). Thromb J.

[2] Connolly SJ, Ezekowitz MD, Yusuf S (2009). Dabigatran versus warfarin in patients with atrial fibrillation. N Engl J Med.

[3] Giugliano RP, Ruff CT, Braunwald E (2013). Edoxaban versus warfarin in patients with atrial fibrillation. N Engl J Med.

[4] Granger CB, Alexander JH, McMurray JJ (2011). Apixaban versus warfarin in patients with atrial fibrillation. N Engl J Med.

[5] Halvorsen S, Ghanima W, Fride Tvete I (2017). A nationwide registry study to compare bleeding rates in patients with atrial fibrillation being prescribed oral anticoagulants. Eur Heart J Cardiovasc Pharmacother.

[6] Hohnloser SH, Basic E, Hohmann C, Nabauer M (2018). Effectiveness and safety of non-vitamin K oral anticoagulants in comparison to phenprocoumon: data from 61,000 patients with atrial fibrillation. Thromb Haemost.

[7] Martinez CAA, Lanas F, Radaideh G (2018). XANTUS-EL: a real-world, prospective, observational study of patients treated with rivaroxaban for stroke prevention in atrial fibrillation in Eastern Europe, Middle East, Africa and Latin America. Egypt Heart J.

[8] Maura G, Billionnet C, Drouin J, Weill A, Neumann A, Pariente A (2019). Oral anticoagulation therapy use in patients with atrial fibrillation after the introduction of non-vitamin K antagonist oral anticoagulants: findings from the French healthcare databases, 2011–2016. BMJ Open.

[9] Mazurek M, Huisman MV, Rothman KJ (2017). Regional differences in antithrombotic treatment for atrial fibrillation: insights from the GLORIA-AF phase II registry. Thromb Haemost.

[10] Mueller T, Alvarez-Madrazo S, Robertson C, Wu O, Bennie M (2019). Comparative safety and effectiveness of direct oral anticoagulants in patients with atrial fibrillation in clinical practice in Scotland. Br J Clin Pharmacol.

[11] Patel MR, Mahaffey KW, Garg J (2011). Rivaroxaban versus warfarin in nonvalvular atrial fibrillation. N Engl J Med.

[12] Pottegard A, Grove EL, Hellfritzsch M (2018). Use of direct oral anticoagulants in the first year after market entry of edoxaban: a Danish nationwide drug utilization study. Pharmacoepidemiol Drug Saf.

[13] Steinberg BA, Gao H, Shrader P (2017). International trends in clinical characteristics and oral anticoagulation treatment for patients with atrial fibrillation: results from the GARFIELD-AF, ORBIT-AF I, and ORBIT-AF II registries. Am Heart J.

[14] Vinogradova Y, Coupland C, Hill T, Hippisley-Cox J (2018). Risks and benefits of direct oral anticoagulants versus warfarin in a real world setting: cohort study in primary care. BMJ.

[15] European Medicines Agency. Lixiana: EPAR—product information. 2015. https://www.ema.europa.eu/en/documents/product-information/lixiana-epar-product-information_en.pdf. Accessed 28-08-2019.

[16] De Caterina R, Kelly P, Monteiro P (2019). Characteristics of patients initiated on edoxaban in Europe: baseline data from edoxaban treatment in routine clinical practice for patients with atrial fibrillation (AF) in Europe (ETNA_AF-Europe). BMC Cardiovasc Disord.

[17] De Caterina R, Kelly P, Monteiro P (2019). Design and rationale of the edoxaban treatment in routine clinical practice for patients with atrial fibrillation in Europe (ETNA-AF-Europe) study. J Cardiovasc Med.

[18] Hanemaaijer S, Sodihardjo F, Horikx A (2015). Trends in antithrombotic drug use and adherence to non-vitamin K oral anticoagulants in the Netherlands. Int J Clin Pharm.

[19] van den Heuvel JM, Hovels AM, Buller HR, Mantel-Teeuwisse AK, de Boer A, Maitland-van der Zee AH (2018). NOACs replace VKA as preferred oral anticoagulant among new patients: a drug utilization study in 560 pharmacies in the Netherlands. Thromb J.

[20] Moudallel S, Steurbaut S, Cornu P, Dupont A (2018). Appropriateness of DOAC prescribing before and during hospital admission and analysis of determinants for inappropriate prescribing. Front Pharmacol.

[21] Santos J, Antonio N, Rocha M, Fortuna A (2020). Impact of direct oral anticoagulants off-label doses on clinical outcomes of atrial fibrillation patients: a systematic review. Br J Clin Pharmacol.

[22] Arbel R, Sergienko R, Hammerman A (2019). Effectiveness and safety of off-label dose-reduced direct oral anticoagulants in atrial fibrillation. Am J Med.

[23] Steinberg BA, Shrader P, Pieper K (2018). Frequency and outcomes of reduced dose non-vitamin K antagonist anticoagulants: results from ORBIT-AF II (the outcomes registry for better informed treatment of atrial fibrillation II). J Am Heart Assoc.

[24] Yao X, Shah ND, Sangaralingham LR, Gersh BJ, Noseworthy PA (2017). Non-vitamin K antagonist oral anticoagulant dosing in patients with atrial fibrillation and renal dysfunction. J Am Coll Cardiol.

[25] Ruff CT, Giugliano RP, Braunwald E (2015). Association between edoxaban dose, concentration, anti-factor Xa activity, and outcomes: an analysis of data from the randomised, double-blind ENGAGE AF-TIMI 48 trial. Lancet.

[26] Stacy ZA, Call WB, Hartmann AP, Peters GL, Richter SK (2016). Edoxaban: a comprehensive review of the pharmacology and clinical data for the management of atrial fibrillation and venous thromboembolism. Cardiol Ther.

[27] Kirchhof P, Benussi S, Kotecha D (2016). 2016 ESC guidelines for the management of atrial fibrillation developed in collaboration with EACTS. Eur Heart J.

[28] Steffel J, Verhamme P, Potpara TS (2018). The 2018 European heart rhythm association practical guide on the use of non-vitamin K antagonist oral anticoagulants in patients with atrial fibrillation. Eur Heart J.

[29] Tjin-A-Tsoi TBPM. The Netherlands on a European scale 2019. 2019. https://longreads.cbs.nl/european-scale-2019/. Accessed 28 Feb 2020.

