# Supplementary material for: Characteristics of patients with atrial fibrillation prescribed edoxaban in Belgium and the Netherlands: insights from the ETNA-AF-Europe study
Source: Neth Heart J. 2021 Jan 7;29(3):158–67. doi: 10.1007/s12471-020-01518-7 (PMC7904979; doi:10.1007/s12471-020-01518-7)
Supplement: Supplementary file 1 — Principal Investigators of ETNA-AF-Europe from Belgium and the Netherlands [file 12471_2020_1518_MOESM1_ESM.pdf]

Supplementary Information of "Characteristics of patients with atrial fibrillation prescribed edoxaban in Belgium and the Netherlands: insights from the ETNA-AF-Europe study"

Principal Investigators of ETNA-AF-Europe

Surname	Initials	Hospital	City
Belgium			
Alzand	B	General Hospital Glorieux Ronse	Ronse
Asmundis, de	C	University Hospital Brussels	Brussels
Baetsle	P-E	General Hospital Geel	Geel
Blankoff	I	Civil Hospital Marie Curie	Charleroi
Boussy	T	General Hospital Groeninge Kortrijk	Kortrijk
Buysschaert	I	General Hospital Aalst	Aalst
Caenepeel[a]	A	N/A	Willebroek
Calozet[b]	Y	N/A	Gribomont
Capiau[b]	L	N/A	Wetteren
Casado Arroyo	R	Hospital Erasme	Anderlecht
Catez	E	Brugmann University Hospital	Brussels
Chevalier	S	Hospital Center Ottignies	Ottignies
Cools	F	General Hospital Klinieken Noord-Antwerpen	Brasschaat
Dierickx[b]	R	N/A	Westouter
Dujardin	K	General Hospital Delta Roeselare	Roeselare
Evrard	P	MontLegia Clinic	Liège
Faes	D	Hospital Maria North-Limburg	Overpelt
Gazagnes	M-D	Brugmann University Hospital	Brussels
Goethals	P	Clinic Saint Jean Brussels	Brussels
Hausman	P	Grand Hopital de Charleroi Charleroi	Gilly
Heidbüchel	H	University Hospital Antwerpen	Edegem
Hemelsoet[c]	D	University Hospital Gent	Ghent
Hoffer	E	University Hospital Gent	Liège
Hollanders[a]	G	N/A	De Pinte
Klippel, de[c]	N	Hospital Jessa Hasselt	Hasselt
Mairesse	GH	Cliniques du Sud-Luxembourg	Arlon
Mamaar	R	Regional Hospital Center Huy	Huy
Man, de	F	Europe Hospitals' Sainte Elisabeth Brussels	Brussels

Paparella	G	Europe Hospitals' Sainte Elisabeth Brussels	Brussels
Pauw, de	M	Ghent University Hospital	Ghent
Potter, de	T	Hospital OLVZ Aalst	Aalst
Put	P	General Hospital Sint-Fransciscus Heusden-Zolder	Heusden-Zolder
Rivero	M	Hospital Oost-Limburg Genk	Genk
Roelandt	R	Hospital Jan Yperman Ieper	Ypres
Rossenbacker	T	General Hospital Imelda Bonheiden	Bonheiden
Sarens	T	General Hospital Sint-Blasius Dendermonde	Dendermonde
Thoeng	J	General Hospital Turnhout	Turnhout
Vanacker[c]	P	General Hospital Groeninge	Kortrijk
Vanassche	T	Leuven University Hospital	Leuven
Vandekerckhove	H	General Hospital Sint-Lucas Gent	Ghent
Vandekerckhove	Y	General Hospital Sint-Jan Brugge	Bruges
Vanstechelman	F	General Hospital Sint-Elisabeth Zottegem	Zottegem
Verleyen	D	General Hospital Sint-Lucas Assebroek	Bruges
Verstraete	S	General Hospital Zeno Knokke-Heist	Knokke-Heist
Vervoort	G	General Hospital Sint-Maarten Mechelen	Mechelen
Vijgen	J	Hospital Jessa Hasselt	Hasselt
Vileyn[b]	G	N/A	Blankenberge
Voet	J	General Hospital Nikolaas Sint-Niklaas	Sint-Niklaas
Xhaet	O	UCL University Clinics of Mont-Godinne	Yvoir
Yperzeele[c d]	L	University Hospital Antwerp	Antwerp
The Netherlands			
Alblas[c]	CL	Franciscus Vlietland	Schiedam
Beelen	DPW	IJsselland Hospital	Capelle aan den IJssel
Berg, ten	J	St. Antonius Hospital	Nieuwegein
Brouwer	MA	Radboud University Medical Centre	Nijmegen
Creanza	M	Rivas Beatrix Hospital	Gorinchem
Crijns	HJGM	Maastricht University Medical Centre+	Maastricht
Driel, van	V	Haga Hospital, Location Sportlaan	The Hague
Groot, de	JR	Amsterdam Medical Centres, Location AMC	Amsterdam
Hartog, den	F	Gelderse Vallei Hospital	Ede
Hemels	MEW	Rijnstate Hospital	Arnhem
Hofstra	JH	Queen Beatrix Hospital	Winterswijk
Holt, ten	W	Amstelland Hospital	Amstelveen
Hoogslag	P	Diaconessenhuis Hospital, Location Meppel	Meppel
Jansen	WPJ	Tergooi hospital	Hilversum
Kuijper	A	Spaarne Hospital	Hoofddorp
Nes, van	E	Laurentius Hospital	Roermond
Nierop	P	Franciscus Gasthuis	Rotterdam

Nooijen, van	F	Dijklander Hospital, Location Purmerend	Purmerend
Nooijer, de	C	Maxima Medical Center	Veldhoven
Prins	F	Elkerliek Hospital, Location Helmond	Helmond
Rasoul	S	Zuyderland Medical Center, Location Heerlen	Heerlen
Spierenburg	H	Franciscus Vlietland	Schiedam
Viergever	E	Groene Hart Hospital	Gouda
Wal, van de	R	Bernhoven Uden	Uden
Westendorp	I	Rode Kruis Hospital	Beverwijk
Wetering, van de	M	BovenlJ Hospital	Amsterdam
Wu	KW	Van Weel-Bethesda Hospital	Dirksland

Names are in alphabetical order. All principal investigators were affiliated with a Department of Cardiology unless stated otherwise.

N/A not applicable; *OLVZ* Onze Lieve Vrouwziekenhuis; *UCL* Université catholique de Louvain; *AMC* Amsterdam Medical Centre

[a] Private Practice in Cardiology; [b] General Practitioner; [c] Department of Neurology; [d] Department of Neurosurgery.
